# Epidemiological aspects of leptospirosis in Rio Grande do Sul during the 2024 Flood

**DOI:** 10.1590/0037-8682-0426-2024

**Published:** 2025-08-18

**Authors:** Renan Konig Leal, Millena Fernandes, Caroline Pereira Vieira, Ester Cristina Corrêa da Rocha, Nícolas Firmiano Flores, Wellyngton Vieira Eufrazio, Carolina Zomer da Silva, Betine Pinto Moehlecke Iser, Josiane Somariva Prophiro

**Affiliations:** 1 Universidade do Sul de Santa Catarina (UNISUL), Programa de Pós-graduação em Ciências da Saúde (PPGCS), Tubarão, SC, Brasil.; 2 Universidade do Sul de Santa Catarina (UNISUL), Curso de Medicina Humana, Tubarão, SC, Brasil.; 3 Instituto Federal de Santa Catarina (IFSC), Programa de Pós-graduação em Clima e Meio Ambiente (PPGCA), Florianópolis, SC, Brasil.

**Keywords:** *Leptospira* infection, Floods, Climate change, Epidemiological monitoring, Epidemiology, Zoonosis surveillance

## Abstract

**Background:**

Leptospirosis is a serious zoonosis. In 2024, Rio Grande do Sul (RS) experienced floods affecting 93% of the municipalities and 8.8% of the population. In this study, we reviewed the leptospirosis notifications post-flooding.

**Methods:**

Descriptive quantitative study using secondary data from the RS Health Department Epidemiological Report.

**Results:**

A total of 7,129 suspected cases were reported, of which 788 were confirmed. Porto Alegre had the highest number of cases and Travesseiro had the highest incidence rate. Epidemiological weeks 20-24 peaked in cases and deaths (47 total cases), with significant municipal variation.

**Conclusions:**

The 2024 floods caused a significant increase in leptospirosis, underscoring the need for a One Health approach and strengthening public health policies.

Leptospirosis is a zoonosis that presents with different levels of seriousness and is caused by bacteria of the genus *Leptospira*. It is transmitted by contact of the skin with the urine of infected animals or contaminated soil and water. Leptospirosis affects both developed and developing countries; in developed countries, it is associated with outdoor activities and in developing countries, with sanitation problems^1^. Climate change increases the risk of transmission, particularly in vulnerable communities. Environmental factors, such as high temperatures and intense rainfall, also influence the viability of *leptospira*; the disease is frequently associated with outbreaks in tropical regions^2^.

Between April and May 2024, the state of Rio Grande do Sul (RS) was affected by a high volume of rainfall, affecting 93% of the 497 municipalities in the state and approximately 8.8% of the state's population and households^3^. Approximately 310,400 vulnerable people and 138,800 families were directly affected by floods and landslides. In this scenario, epidemiological surveillance and public management should be aware of the health impacts of such conditions on people and animals, such as an increase in the occurrence of diseases, including leptospirosis^3^.

Accurate estimates of the local distribution of leptospirosis are essential to guide policies and raise community awareness. Monitoring the short-term evolution of disease in vulnerable areas can aid in the formulation, adaptation, and mitigation of disease control strategies. The present study aimed to review the notifications of leptospirosis in RS after flooding in 2024.

Descriptive and quantitative study with secondary data from the Epidemiological Report (ER) issued by the Health Department of the State of RS and released on August 15, 2024^4^, whose tabulation included data collected from April 26 to August 15, 2024. 

This Epidemiological Report included variables such as the number of cases, cases reported by the municipality of residence, deaths, and data in each Epidemiological Week (EW). To complement the data from the RS Health Department, the incidence, mortality, and lethality indicators were calculated based on population data reported by the *Instituto Brasileiro de Geografia e Estatística* (IBGE; Brazilian Institute of Geography and Statistics). The Epidemiological Report includes notifications by the municipality but does not indicate confirmed cases. Therefore, confirmed leptospirosis data retrieved from the *Sistema de Informação de Agravos de Notificação* (SINAN, Notifiable Diseases Information System) from April to August 2024, the same period as in the aforementioned Epidemiological Report, were used.

To calculate the annual variation in the average number of leptospirosis cases per epidemiological week, the months corresponding to the same epidemiological weeks as in the Epidemiological Report for the years 2021, 2022, 2023, and 2024 were selected from the SINAN Report. To calculate the rate of disease growth relative to the previous year, the initial rate was subtracted from the final rate, divided by the initial rate, and multiplied by 100.

In addition, a thematic map of the geospatial distribution and incidence of confirmed leptospirosis cases in RS was developed for the study period using a Geographic Information System (GIS). Disease incidence intervals were set to portray the risk of leptospirosis in the different RS municipalities. Four intervals were defined: *no information available, low risk* (0.1 to 4.99), *risk alert* (5.00 to 8.99), and *high risk* (9.00+). This categorization was adopted to avoid the loss of important information that could occur within wider ranges, such as those based on natural breaks or quantiles. The cutoff values, specifically 4.99 and 8.99, were set to clearly highlight the differences in risk and warning for the areas of greatest concern.

During the review period, 7,129 suspected cases of leptospirosis were reported in the RS. Of these, 788 (11.1%) were confirmed, 3,497 (49.1%) were discarded, and 2,844 (39.9%) were still under investigation. The analysis of the case distribution reported by the municipality of residence revealed that Porto Alegre had the highest number of cases reported, totaling 1,959 cases. The municipality of Canoas was second with 647 reported cases, followed by São Leopoldo with 409 cases, Alvorada with 313 cases, Novo Hamburgo with 299 cases, Sapucaia do Sul with 264 cases, Santa Cruz do Sul with 182 cases, Igrejinha with 166 cases, and Viamão with 138 cases.

The analysis of leptospirosis incidence rates revealed significant variations among municipalities. Although not included in the list of notifications in the Epidemiological Report mentioned above, the following municipalities proved to be relevant for the incidence analysis based on the SINAN data. Travesseiro had the highest rate, reporting 139.4 cases per 100,000 inhabitants, followed by Três Coroas with 131.0 per 100,000 inhabitants. Other municipalities such as Mato Leitão and Santo Antônio do Palma had rates of 102.9 and 95.6 per 100,000 inhabitants, respectively. Smaller municipalities such as Colinas and Maratá recorded rates of 82.5 and 80.9, respectively. In addition, municipalities such as Cruzeiro do Sul, Charrua, Montauri, Nova Palma, and Ibarama had rates ranging from 77.5 to 53.5. Additionally, other municipalities reported a significant incidence, as illustrated in Figure 1.

**FIGURE 1:**
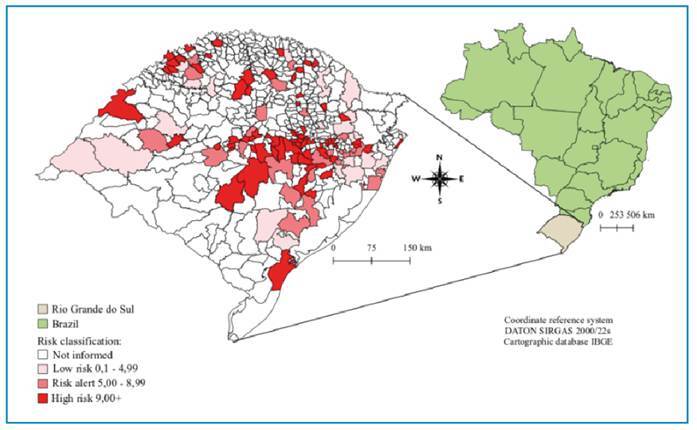
Risk classification of municipalities in Rio Grande do Sul according to the incidence rate.

Analysis of leptospirosis data according to epidemiological week (EW) from EW 20 (May 12, 2024) to EW 33 (August 17, 2024) revealed significant variations in the number of reported cases and deaths (Figure 2). In EW 20, 659 cases were reported, with 8 recorded deaths. In EW 21, there was an increase to 1088 cases with 7 deaths, and this upward trend continued in EW 22 with 1129 cases and 6 deaths. The peak in notifications occurred at EW 23, with 1174 cases and no reported deaths. EW 24 saw a slight decrease to 1071 cases, but an increase in deaths to 8. By EW 25, the number of cases had declined to 616, with only 1 death.

**FIGURE 2:**
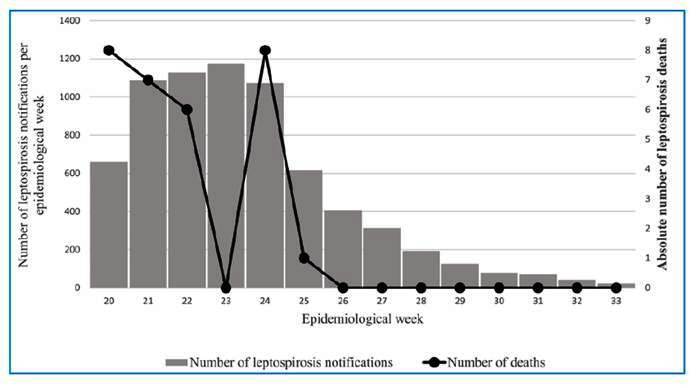
Number of leptospirosis cases per epidemiological week.

In the subsequent weeks, there was a continued decline. EW 26 recorded 404 cases with no deaths, followed by 313 cases in EW 27, and 194 cases in EW 28, with no deaths in either week. By EW 33, the number of cases had decreased significantly to 22, with no recorded deaths. These data suggest a high incidence of leptospirosis between weeks 20 and 24, followed by a steady decline in cases, with deaths ceasing from EW 26 onward.

When comparing the average number of leptospirosis cases for the same epidemiological weeks of the study in the years 2021 to 2024, a significant growth was observed (Figure 3). In fact, in 2021, the average was 12.8 cases, rising to 24.6 in 2022, increasing further to 28.8 in 2023; in 2024, the average soared to 73.6 cases, indicating an abrupt and unexpected increase. From 2021 to 2022, there was an increase of 92.19%; from 2022 to 2023, the increase was 17.07%; and from 2023 to 2024, the growth was 155.56%.

**FIGURE 3:**
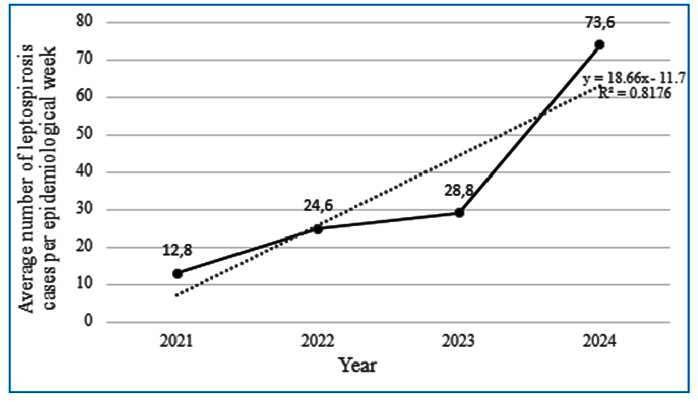
Average number of leptospirosis cases per epidemiological week per year.

In addition to the above, 47 deaths were reported, of which 26 were confirmed to result from leptospirosis, with four cases still under investigation and 17 discarded. Analysis of the distribution according to sex and age group showed that most deaths involved men aged 50-59 years (10 deaths), followed by men aged 60-69 years (5 deaths). Deaths were also recorded in other age groups, including two in individuals under 29 years of age, three between 30 and 39 years of age, four between 40 and 49 years of age, and one between 70 and 79 years of age, with no records of deaths in individuals aged 80 years or older. One woman aged 60-69 years was confirmed among the deaths caused by leptospirosis. 

An analysis of leptospirosis mortality and lethality rates across municipalities in the RS revealed significant regional variations. Alecrim had mortality rate of 16.33 and a lethality rate of 100%. In Alvorada, the rates were 1.07 for mortality and 20% for lethality, while Cachoeirinha had a mortality rate of 0.73 and a high lethality rate of 100%. Canoas had one of the lowest rates, with a mortality of 0.29 and lethality of 3.7%.

Capela de Santana had a mortality rate of 8.96 and lethality of 100%, while Charqueadas recorded a mortality rate of 2.86 and a lethality of 25%. Encantado had one of the highest mortality rates, with a mortality of 22.37 and a lethality rate of 100%. In Estrela and Guaíba, the mortality rates were 3.11 and 1.08, respectively, both with a lethality rate of 9.09%. Harmonia had a mortality rate of 18.59 and lethality of 100%. In Igrejinha, the rates were 3.05 for mortality and 7.14% for lethality, while Novo Hamburgo registered a mortality rate of 0.88 and a lethality of 11.11%. In Pelotas, the mortality was 0.31 and the lethality 20%, whereas Porto Alegre had a mortality of 0.30 and lethality of 9.76%. In Rio Grande, the mortality rate was 0.52 and lethality was 3.85%.

São Leopoldo recorded a mortality rate of 0.32 and lethality of 12.5%, whereas Sapucaia do Sul had a mortality rate of 0.76 and lethality of 33.33%. Travesseiro had one of the highest rates, with a mortality of 46.47 and lethality of 33.33%. Três Coroas had a mortality rate of 4.09 and lethality of 3.13%, while Venâncio Aires showed a mortality rate of 1.45 and lethality of 4.76%. Finally, Viamão registered a mortality rate of 0.45 and lethality of 12.5%.

The results revealed a considerable increase in leptospirosis cases in RS for epidemiological weeks 20 to 32 of the year 2024. Growth had already been observed, but to a lesser extent, in previous years (2021, 2022, 2023). In 2021, there were no confirmed cases. In 2022, 98 cases were confirmed, and in 2023, 117 cases were confirmed. In 2024, there is an increase of 788 confirmed cases, and 3,497 cases are under investigation. This growth reflects the impact of floods with water polluted with rodent feces and urine contaminated with *Leptospira*, increasing the risk of infection^5^. These data corroborate the epidemiological situation of leptospirosis in the southern and southeastern regions of Brazil, where there are high rates of cases and deaths^6^, which can be intensified by extreme weather events such as floods^5^.

The spatial distribution of leptospirosis in the RS showed that the municipality of Porto Alegre had the highest number of reported cases, with 1,959 cases. The municipality has 5.2% of its population living in areas with open sewage, 11.8% in regions without sewage or water and sewage systems, and 6.0% with garbage accumulation in the streets, although garbage collection is conducted in 100% of the city^7^.

Mortality results showed that men aged 50-59 years were the most affected by leptospirosis, a finding similar to other studies that indicated a higher number of deaths recorded in men^8^ and older adults^9^. Although occupational exposure to infected animals and floodwater has not been directly evaluated in the confirmed cases and deaths, such exposure is relevant to the epidemiological context of the disease.

In line with the above, a "One Health" approach is proposed in the epidemiological context of zoonotic infections such as leptospirosis, seeking integration across multiple sectors for early detection of outbreaks and a successful response, aiming to achieve optimal health for people, animals, and the environment^10^.

This study highlights leptospirosis as a public health problem in RS in 2024, which is exacerbated by floods. The significant increase in confirmed cases and the high incidence in some municipalities indicates the need to intensify preventive and control measures. The high fatality rate among men aged 50-59 years highlights the urgency of campaigns targeting at-risk groups. The "One Health" approach integrates human, animal, and environmental health, allowing a more effective response to zoonotic risks related to climate change. Enhancing epidemiological surveillance and implementing public policies to mitigate the effects of natural disasters are recommended, thereby contributing to a reduction in leptospirosis cases.
